# Comparative study of ternary hybrid nanofluids with role of thermal radiation and Cattaneo-Christov heat flux between double rotating disks

**DOI:** 10.1038/s41598-023-34783-8

**Published:** 2023-05-13

**Authors:** Sobia Noreen, Umar Farooq, Hassan Waqas, Nahid Fatima, M. S. Alqurashi, Muhammad Imran, Ali Akgül, Abdul Bariq

**Affiliations:** 1 Department of Chemistry, Government College Women University, Faisalabad, 38000 Pakistan; 2 Department of Mathematics, Government College University, Faisalabad, 38000 Pakistan; 3 School of Energy and Power Engineering, Jiangsu University, Zhenjiang, 212013 China; 4 Department of Mathematics and Sciences, Prince Sultan University, Riyadh, 11586 Saudi Arabia; 5 Department of Mathematics, College of Science, Taif University, P. O. Box 11099, Taif, 21944 Saudi Arabia; 6 Department of Computer Science and Mathematics, Lebanese American University, Beirut, Lebanon; 7 Department of Mathematics, Art and Science Faculty, Siirt University, 56100 Siirt, Turkey; 8 Department of Mathematics, Mathematics Research Center, Near East University, Near East Boulevard, PC: 99138 Nicosia/Mersin 10, Turkey; 9 Department of Mathematics, Laghman University, MehPPapetarlam 2701, Laghman, Afghanistan

**Keywords:** Mathematics and computing, Nanoscience and technology, Physics

## Abstract

Heat and mass transfer are crucial to numerous technical and commercial operations, including air conditioning, machinery power collectors, crop damage, processing food, heat transfer mechanisms, and cooling, among numerous others. The fundamental purpose of this research is to use the Cattaneo-Christov heat flux model to disclose an MHD flow of ternary hybrid nanofluid through double discs. The results of a heat source and a magnetic field are therefore included in a system of PDEs that model the occurrences. These are transformed into an ODE system using similarity replacements. The first-order differential equations that emerge are then handled using the computational technique Bvp4c shooting scheme. The Bvp4c function in MATLAB is used to numerically solve the governing equations. The influence of the key important factors on velocity, temperature, nanoparticles concentration, and is illustrated visually. Furthermore, increasing the volume fraction of nanoparticles improves thermal conduction, increasing the heat transfer rate at the top disc. The graph indicates that a slight increase in melting parameter rapidly declines the velocity distribution profile of nanofluid. The temperature profile was boosted due to the growing outcomes of the Prandtl number. The increasing variations of the thermal relaxation parameter decline the thermal distribution profile. Furthermore, for some exceptional instances, the obtained numerical answers were compared to previously disclosed data, yielding a satisfactory compromise. We believe that this discovery will have far-reaching ramifications in engineering, medicine, and the field of biomedical technology. Additionally, this model can be used to examine biological mechanisms, surgical techniques, nano-pharmacological drug delivery systems, and the therapy of diseases like cholesterol using nanotechnology.

## Introduction

In many engineering systems, such as fuel cells, heat exchangers, and others, fluids play a crucial role in accelerating the rate of heat transfer. However, due to the weak heat conductivity of normal fluids, we need specialized, high-thermal conductivity fluids to solve this issue. Choi^1^ proposed the term nanofluid for the first time. The major feature of nanofluids is that they have better thermal conductivity than ordinary fluids due to metallic nanometer-sized particles floating in fluid, which significantly contribute to thermal conductivity enhancement. Many researchers have concentrated their efforts on fluid flow and heat transfer topics, whether employing nanofluid or ordinary fluid. Using a hybrid nanofluid flow over a rotating surface, Seed et al.^2^ investigated the effects of entropy production. The electromagnetic flow of hybrid nanofluids traveling across two discs was studied by Farooq et al.^3^. Agrawal and Kaswan^4^ looked at the effects of entropy production on the flow of hybrid nanofluids through discs. In the context of heat electromagnetic radiation, Abbas et al.^5^ looked into the Marangoni flow of a mixed nanofluid through a disc. In the context of a modified Cattaneo-Christov flux and heat source, Farooq et al.^6^ investigated nanofluid flow was conducted. Nanofluids were studied by Li et al.^7^ utilizing heat radiation and movable microorganisms. Madhukesh et al.^8^ investigated the thermal properties of ternary hybrid nanofluid flow across a cylinder/plate. Arif et al.^9^ looked at the thermal consequences of a nanofluid radiative flow across a rotating disc. In mixed convection, Patil and Goudar^10^ investigated the impact of nanofluid flow. The effects of the heat transfer assessment of a fractional couple stress model and Casson tri-hybrid nanofluid in blood with different types of nanoparticles were examined by Arif et al.^11^.

In terms of energy exchange, ternary hybrid nanofluid outperforms ordinary fluids, nanofluid, hybrid nanofluid, gasoline, and methanol. High-temperature freezing is one of the thermodynamic repercussions of hybrid nanofluids. Solar energy, heating and cooling systems, heat exchangers, purification systems for the automobile sector, electrical chillers, turbines, nuclear networks, broadcasters, ships, and biotechnology all employ hybrid nanofluids. Various non-Newtonian theories for ternary hybrid nanostructures were evaluated by Nazir et al.^12^. The influence of suction and heat source on ternary hybrid nanofluid MHD flow through a cylinder was explored by Mahmood et al.^13^. Sajid et al.^14^ looked at the chemical processes that occur when Arrhenius energy is applied to ternary hybridity nanofluids over a wedge. Khan et al.^15^ investigated the flow of ternary nanoparticles that are hybrids over a sphere. In the context of thermal augmentation programs, Ullah^16^ explored the Marangoni effects of tri-hybrid nanoflow. Saleem et al.^17^ investigated the consequences of thermally radiated brain matter-based ternary hybrid nanofluid. Alwawi et al.^18^ investigated the effect of ternary nano-compositions on liquid heat exchange efficiency. Algehyne et al.^19^ studied the consequences of Cu-based hybrid nanofluid flow through a porous cavity. Dovom et al.^20^ examined the computational study of the heating aerosol's carbon nanofluid flow in an electrical plant. The efficacy development of nanofluid hybrid flow on a spinning disc was studied by Upreti and Mishra^21^. using double Cattaneo-Christov diffusion. The effects of stress, flow, and heat transfer over a spinning disc with temperature-dependent nanofluid characteristics were examined by Upreti et al.^22^. Upreti et al.^23^ studied the MHD flow rate of nanofluid across a porous surface using the Xue framework. Pandey et al.^24^ looked at the impact of spontaneous convection on the three-dimensional flow of nanostructures across porous surfaces.

Magnetization is an essential element in manufacturing as well as engineering, with several applications. The chemical reactions of fluid nanocrystals with magnetic fields influence the quality of heat transfer, clutches, and compressors that are available among various industrial items. Interplanetary and astrophysical magnetospheres, as well as aerospace and chemical science, make use of magnetic fields. The flow properties are influenced by the strength and dispersion of the supplied metamaterials. Many academics have produced fluid mechanics experiments that detail flow properties under the action of a magnetic field. The effects of MHD flow and heat transmission of a hybrid nanofluid across a spinning disc were investigated by Reddy et al.^25^. The MHD flow of hybrid nanofluid technologies with electromagnetic radiation from heat across a spinning disc was investigated by Waqas et al.^26^. Employing a revolving disc, Fallah et al.^27^ controlled the turbulent motion and the heat transfer of a hybrid nanofluid. Usman et al.^28^ study the flow of an unstable spinning disc using a nanofluid composed of a hybrid duo stress film. Heat radiation was employed by Shoaib et al.^29^ to investigate MHD hybrid nanofluids across a rotating disc. The MHD flow phenomena of nanofluid with radiative heat exchange over a rotating, vertical disc with partial slip were studied by Kumar and Sharma^30^. Ramzan et al.^31^ employed solar light to facilitate the slip flow of a hybrid nanofluid over a revolving disc. Gowda et al.^32^ investigated the flow of a nanofluid on a revolving disc that is dependent on time. The effects of a magnetic field on the boundary-layer flow of a Sisko liquid containing nanoparticles moving over a surface were investigated by Khan et al.^33^. The effects of simultaneously creating entropy and solutions in mixed convection stagnation point flow were investigated by Zaib et al.^34^. The effective Prandtl theory on coupled convection flow of nano liquids with micropolar liquid driven by wedge was studied by Zaib et al.^35^. Khan et al.^36^ looked at the radiative stagnation point flow of hybrid nanofluid technology in a cylinder. Nisar et al.^37^ investigated the radiative flow of nanofluid from a curved surface containing blood containing silver nanoparticles.

The present investigation focuses on employing heat radiation to investigate the MHD flow of ternary hybrid nanofluid across double disks. The study also investigates the effects of thermal conductivity and heat source/sink on the transfer of heat parameters. We employ *Fe*_*3*_*O*_*4*_, *Cu*, and *SiO*_*2*_ nanoparticles as the base fluid, together with Kerosene oil. With the help of relevant transformation variables, similarity equations are generated and numerically incorporated in MATLAB employing a built-in method called the shooting method. The amazing properties of physical parameters are explained using graphical representations of the Nusselt number, velocity, and temperature fields. This numerical simulation's findings have been used in spin coating, centrifugal elimination, medical devices, gasoline turbine rotors, and heat power generation systems.

## Mathematical formulation

**Figure 1:**
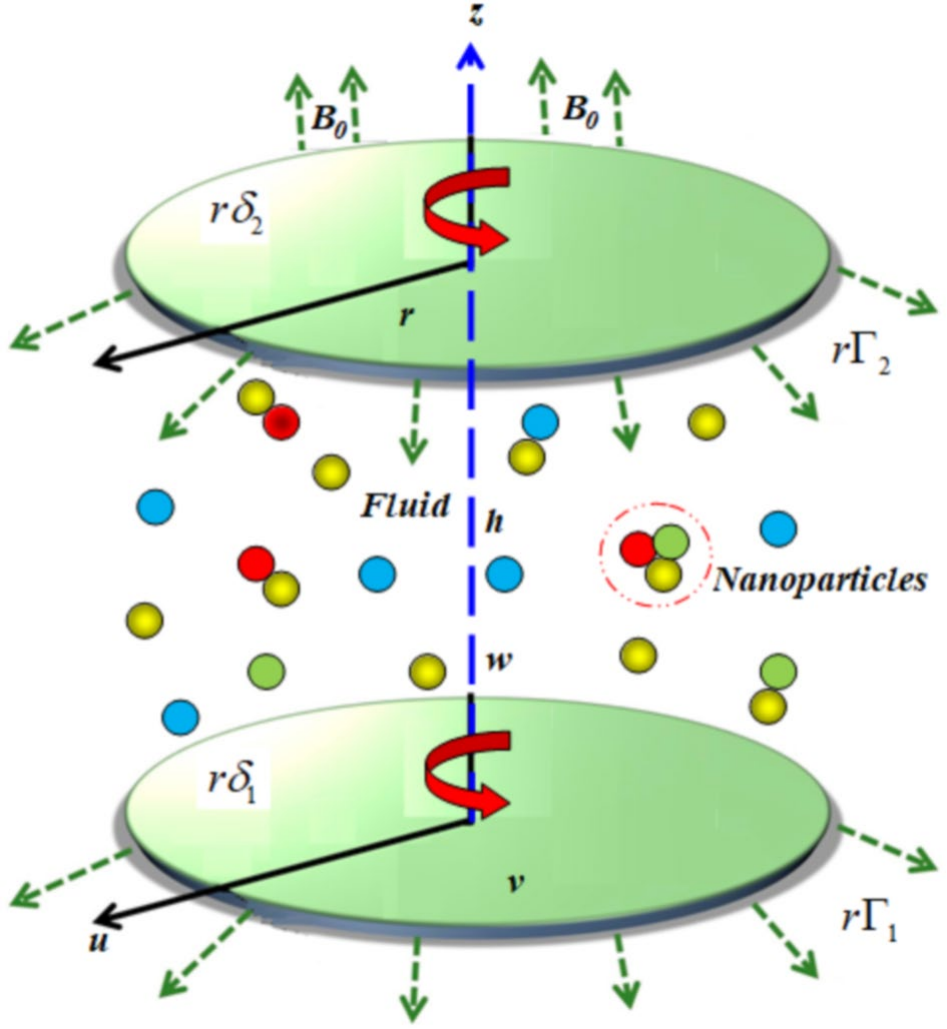
Curve of the flow model.

Here we investigate the effects of ternary hybrid nanofluid passing through a double disk. The Cattaneo-Christov heat flux theory is also taken into the investigation. We use *Fe*_*3*_*O*_*4*_*, Cu,* and *SiO*_*2*_ nanoparticles with Kerosene oil as the base fluid. The Maxwell model is employed to describe non-Newtonian fluid dynamics. Employing the proper similarity transformation, the momentum, and temperature-regulating PDEs are converted into ODEs. Nanofluids are often employed as coolants in heat exchange equipment such as thermal exchange mechanisms, computer cooling mechanisms, and radiators due to their increased thermal characteristics. Many scientists have investigated how heat moves over a double disc. The upper disk rotates with angular velocity $\left( {\Gamma_{1} } \right)$ and the lower disk rotates with the angular velocity $\left( {\Gamma_{2} } \right)$ with a distance of disks $\left( h \right)$ in Fig. 1.

The major findings of the current framework are listed below:

- The steady incompressible, MHD flow is considered for investigation with Kerosene oil as base fluid.
- The axisymmetric flow of ternary hybrid nanofluid over a double disk is studied.
- We used the *Fe*_*3*_*O*_*4*_*, Cu,* and *SiO*_*2*_ nanoparticles.
- The importance of the Cattaneo-Christov heat flux theory with thermal radiation is investigated.
- The heat source sink is also taken into consideration.
- Here we used the porous medium for the geometry.
- Here we in analyzed the thermophysical properties of nanoparticles and based fluid.

The key PDEs of the flow problem are^38–40^:

1 $$ u_{r} + \frac{u}{r} + w_{z} = 0, $$

2 $$ \begin{aligned} uu_{r} & - \frac{{v^{2} }}{r} + wu_{z} + \Theta \left( {v^{2} u_{zz} + u^{2} u_{rr} + 2uvu_{rz} } \right) \\ & = - \frac{1}{{\rho_{thnf} }}p_{r} + \frac{{\mu_{thnf} }}{{\rho_{thnf} }}\left[ {u_{rr} + u_{zz} + \frac{1}{r}u_{r} - \frac{u}{{r^{2} }}} \right] \\ & \;\;\; - \frac{{\nu_{thnf} }}{{k^{*} }}u - \frac{{\sigma_{thnf} }}{{\rho_{thnf} }}\beta_{0}^{2} \left( {u + \Theta vu_{z} } \right), \\ \end{aligned} $$

3 $$ \begin{aligned} uv_{r} & + \frac{uv}{r} + wv_{z} + \Theta \left( {v^{2} u_{zz} + u^{2} u_{rr} + 2uvu_{rz} } \right) \\ & = - \frac{1}{{\rho_{thnf} }}p_{r} - \frac{{\mu_{thnf} }}{{\rho_{thnf} }}\left[ {\frac{v}{{r^{2} }} + v_{rr} + \frac{1}{r}v_{r} + v_{zz} } \right] \\ & \;\;\; - \frac{{\nu_{thnf} }}{{k^{*} }}v + \frac{{\sigma_{thnf} }}{{\rho_{thnf} }}\beta_{0}^{2} \left( { - v - \Theta uv_{z} } \right), \\ \end{aligned} $$

4 $$ uw_{r} + ww_{z} = - \frac{1}{{\rho_{thnf} }}p_{z} + \frac{{\mu_{thnf} }}{{\rho_{thnf} }}\left[ {\frac{1}{r}w_{r} + w_{rr} + w_{zz} } \right], $$

5 $$ \begin{aligned} \left( {uT_{r} + wT_{z} } \right) & = \frac{{k_{thnf} }}{{\left( {\rho c_{p} } \right)_{thnf} }}\left[ {T_{zz} + \frac{1}{r}T_{z} + T_{zz} } \right] - \frac{1}{{\left( {\rho c_{p} } \right)_{thnf} }}q_{{r_{z} }} + \frac{{Q_{0} }}{{\left( {\rho cp} \right)_{thnf} \left( {T - T_{\infty } } \right)}} \\ & \;\;\; - \lambda \left[ {u^{2} T_{rr} + 2uwT_{rz} + w^{2} T_{zz} + \left( {wu_{z} + uu_{r} } \right)T_{r} + \left( {ww_{z} + uw_{r} } \right)T_{z} } \right], \\ \end{aligned} $$

With^38–40^

6 $$ \left. \begin{gathered} u\left( { = r\delta_{1} } \right),\,\,\,\,\,\,\,\,v\left( { = r\Gamma_{1} } \right),\,\,\,\,\,\,w\left( { = 0} \right),\,\,\,\,\,\,\,\,\,T\left( { = T_{ \circ } } \right)\,, \hfill \\ at\,\,z\left( { = 0} \right), \hfill \\ u\left( { = r\delta_{2} } \right),\,\,\,\,\,\,\,v\left( { = r\Gamma_{2} } \right),\,\,\,\,\,\,\,w\left( { = 0} \right),\,\,\,\,\,\,\,\,T\left( { = T_{1} } \right), \hfill \\ at\,\,z\left( { = h} \right), \hfill \\ k_{thnf} \left( {T_{z} } \right)_{z = 0} \left( { = \rho_{thnf} \left[ {L_{ \circ } + \left( {c_{p} } \right)_{s} \left( {T_{ \circ } - T_{1} } \right)} \right]w} \right) \hfill \\ \end{gathered} \right\}. $$

Here the viscosity of hybrid nanofluid $\left( {\mu_{hnf} } \right)$, the thermal conductivity of nanofluid is denoted by $\left( {K_{nf} } \right)$, the density of hybrid nanofluid is $\left( {\rho_{hnf} } \right)$, the viscosity of nanofluid $\left( {\mu_{nf} } \right)$, the thermal conductivity of hybrid nanofluid is denoted by $\left( {K_{hnf} } \right)$, the heat capacity of ternary hybrid nanofluid is $\left( {\rho C_{p} } \right)_{thnf}$, the heat capacity of hybrid nanofluid is $\left( {\rho C_{p} } \right)_{hnf}$, the viscosity of ternary hybrid nanofluid $\left( {\mu_{thnf} } \right)$, the heat capacity of nanofluid is $\left( {\rho C_{p} } \right)_{nf}$, the thermal conductivity of ternary hybrid nanofluid is denoted by $\left( {K_{thnf} } \right)$, the density of nanofluid is $\left( {\rho_{nf} } \right)$, and density of ternary hybrid nanofluid is $\left( {\rho_{thnf} } \right)$.

Radioactivity assessment by Roseland The radiation heat flow $\left( {q_{r} } \right)$ is calculated as follows:

7 $$ q_{r} \left( { = - \frac{{4\sigma^{*} }}{{3K^{*} }}T_{z}^{4} } \right) $$

This is possible by expanding $\left( {T^{4} } \right)$ in the Taylor sequence about the temperature of the free stream $\left( {T_{\infty } } \right)$ as^38^:

8 $$ T^{4} \left( { = T_{\infty }^{4} + 4T_{\infty }^{3} \left( {T - T_{\infty } } \right) + 6T_{\infty }^{2} \left( {T - T_{\infty } } \right)^{2} + \cdot \cdot } \right) $$

In (8) reduce the terms of high order and external the first $\left( {T - T_{\infty } } \right)$ then we acquire:

9 $$ T^{4} \left( {\tilde{ = }4T_{\infty }^{3} T - 3T_{\infty }^{4} } \right) $$

Put (9) into (7) then we obtain:

10 $$ q_{r} \left( { = - \frac{{16T_{\infty }^{3} \sigma^{*} }}{{3K^{*} }}T_{z} } \right) $$

The stream function is $\left( \Psi \right)$:

11 $$ u\left( { = \left( {\Psi_{y} } \right)} \right),\,\,\,v\left( { = \left( { - \Psi_{x} } \right)} \right) $$

The similarities variables are:

12 $$ \left. \begin{gathered} u\left( { = r\Gamma_{1} f^{\prime}\left( \eta \right)} \right),\,v\left( { = r\Gamma_{1} g\left( \eta \right)} \right),w\left( { = - \sqrt {2\nu_{f} \Gamma_{1} } f\left( \eta \right)} \right), \hfill \\ \eta \left( { = \frac{z}{h}} \right),\theta \left( { = \frac{{T - T_{1} }}{{T_{ \circ } - T_{1} }}} \right),\,\,p\left( { = \rho_{f} \Gamma_{1} \nu_{f} \left( {p\left( \eta \right) + \frac{{1r^{2} }}{{2h^{2} }} \in } \right)} \right) \hfill \\ \end{gathered} \right\}. $$

The outcomes of main PDEs into the dimensionless ODEs are:

13 $$ \begin{aligned} f^{\prime\prime\prime} & - \frac{{C_{1} }}{2}{\text{Re}} \left( { - 2ff^{\prime\prime} - g^{2} + f^{{\prime}{2}} } \right) - {\text{Ra}} \beta C_{1} \left( {f^{2} f^{\prime\prime\prime} - 2ff^{\prime\prime}f^{\prime}} \right) - C_{2} P{\text{Re}} f^{\prime} \\ & - \frac{{C_{1} }}{{C_{2} }}Ha\left( {f^{\prime} + \beta f^{\prime\prime}f} \right) - \frac{{C_{1} }}{{C_{2} }} \in = 0, \\ \end{aligned} $$

14 $$ g^{\prime\prime} + C_{1} {\text{Re}} \left( {2fg^{\prime} - 2f^{\prime}g} \right) - C_{2} P{\text{Reg}} - \frac{{C_{1} }}{{C_{2} }}Ha\left( {g + \beta fg^{\prime}} \right) = 0, $$

15 $$ p^{\prime} = 4C_{2} {\text{Re}} ff^{\prime} + 2\frac{{C_{2} }}{{C_{1} }}f^{\prime\prime}, $$

16 $$ \left( {1 + Nr} \right)\theta^{\prime\prime} - 4Q_{T} C_{3} {\text{Re}} \left( {ff^{\prime} + f^{2} \theta^{\prime\prime}} \right) + 2{\text{Re}} {\text{PrC}}_{3} C_{4} f\theta^{\prime} + \Pr Q\theta = 0, $$

The results of boundary conditions

17 $$ \left. \begin{gathered} \eta = 0,\,\,\,\frac{{\left( \rho \right)_{thnf} }}{{\left( \rho \right)_{f} }}\Pr f + Me\frac{{\left( k \right)_{thnf} }}{{\left( k \right)_{f} }}\theta^{\prime},f^{\prime} = s_{1} ,g = 1,\,\theta^{\prime} = 1 \hfill \\ \eta = 1,\,\,\,\,\,\,\,\,\,\,\,\,\,\,\,\,f^{\prime} = s_{2} ,\,\,\,\,\,\,\,\,\,\,\,\,\,\,\,\,\,\,\,\,g = s_{3} ,\,\,\,\,\,\,\,\,\,\,\,\,\,\,\,\,\,\,\,\,\,\theta^{\prime} = 0 \hfill \\ \end{gathered} \right\}. $$

Here

**Table:** 

Mathematical values	Notations	Parameter name
$Ha\left( { = \frac{{\sigma \beta_{0}^{2} }}{{2\Gamma_{1} \rho_{f} }}} \right)$	$\left( {Ha} \right)$	Magnetic parameter
$s_{2} \left( { = \frac{{\delta_{2} }}{{\Gamma_{1} }}} \right)$	$\left( {s_{2} } \right)$	Extending parameter of the upper disk
${\text{Re}} \left( { = \frac{{\Gamma_{1} h^{2} }}{{\nu_{f} }}} \right)$	$\left( {\text{Re}} \right)$	Reynolds number
$Nr\left( { = \frac{{16\sigma^{*} T_{\infty }^{3} }}{{3k_{f} K^{*} }}} \right)$	$\left( {Nr} \right)$	Thermal radiation
$Pr\left( { = \frac{{\nu_{f} }}{{\alpha_{f} }}} \right)$	$\left( {Pr} \right)$	Prandtl number
$Me\left( { = \frac{{\left( {c_{p} } \right)_{f} \left( {T_{1} - T_{1} } \right)}}{{L^{*} + \left( {c_{p} } \right)_{S} \left( {T_{ \circ } - T_{1} } \right)}}} \right)$	$\left( {Me} \right)$	Melting parameter
$s_{1} \left( { = \frac{{\delta_{1} }}{{\Gamma_{1} }}} \right)$	$\left( {s_{1} } \right)$	Extending parameter of the lower disk
$Q = \left( {\frac{{Q_{0} }}{\rho cp}} \right)$	$\left( Q \right)$	Heat source-sink parameter
$Q_{T} \left( { = \Gamma_{1} \lambda } \right)$	$\left( {Q_{T} } \right)$	Thermal relaxation parameter
$\,C_{1} \left( { = \left( {1 - \phi } \right)^{2.5} \left( {1 - \phi + \phi \left( {\frac{{\rho_{s} }}{{\rho_{f} }}} \right)} \right)} \right)$	$\left( {\,C_{1} } \right)$	Constant
$C_{2} \left( { = \left( {1 - \phi } \right) + \phi \left( {\frac{{\rho_{s} }}{{\rho_{f} }}} \right)} \right)$	$\left( {C_{2} } \right)$	Constant
$C_{3} \left( { = \left( {1 - \phi } \right) + \phi \frac{{\left( {\rho C_{p} } \right)_{s} }}{{\left( {\rho C_{p} } \right)_{f} }}} \right)$	$\left( {C_{3} } \right)$	Constant
$C_{4} \left( { = \frac{{k_{nf} }}{{k_{f} }}} \right)$	$\left( {C_{4} } \right)$	Constant
$s_{3} \left( { = \frac{{\Gamma_{1} }}{{\Gamma_{2} }}} \right)$	$\left( {s_{3} } \right)$	Rotation parameter

From (13) differentiated for $\left( \zeta \right)$ and eradicate $\left( \Gamma \right)$ then we obtain:

18 $$ \begin{aligned} f^{iv} & + C_{1} {\text{Re}} \left( \begin{gathered} ff^{\prime\prime\prime} \hfill \\ + gg^{\prime} \hfill \\ \end{gathered} \right) - C_{2} P{\text{Re}} f^{\prime\prime} + {\text{Re}} \beta C_{1} \left( {f^{2} f^{\prime\prime\prime} + 2f^{\prime\prime}f^{{\prime}{2}} + 2ff^{{\prime\prime}{2}} } \right) \\ & \, - \frac{{C_{1} }}{{C_{2} }}Ha\left( {f^{\prime\prime} + \beta f^{\prime}f^{\prime\prime} + \beta ff^{\prime\prime\prime}} \right) = 0, \\ \end{aligned} $$

From (13 and 17) the constraints $\left( \in \right)$ which demonstrate pressure as:

19 $$ \begin{aligned} \in & = \frac{{C_{2} }}{{C_{1} }}f^{\prime\prime\prime} - C_{2} P{\text{Re}} f^{\prime\prime} - \frac{{C_{2} }}{2}{\text{Re}} \left[ {f^{{\prime}{2}} - 2ff^{\prime\prime} - g^{2} } \right] \\ & \;\;\; - {\text{Re}} \beta C_{2} \left[ {f^{2} f^{\prime\prime\prime} - 2ff^{\prime}f^{\prime\prime}} \right] - Ha\left( {f^{\prime} + \beta f^{\prime}f^{\prime\prime}} \right) \\ \end{aligned} $$

From (15) integrated to $\left( \zeta \right)$ by using following limits $\left( {0\,\,\,\,\,to\,\,\,\,\eta } \right)$ to get pressure term.

20 $$ p + 2C_{2} {\text{Re}} f^{2} + 2\frac{{C_{2} }}{{C_{1} }}\left( {f^{\prime} - f^{\prime}} \right) = 0, $$

The engineering parameters for both disks are skin friction $\left( {\left( {C_{f} } \right)_{1} \;{\text{and}}\;\left( {C_{f} } \right)_{2} } \right)$ and Nusselt numbers $\left( {\left( {Nu_{x} } \right)_{1} \;{\text{and}}\;\left( {Nu_{x} } \right)_{2} } \right)$.

21 $$ \left. \begin{gathered} \left( {C_{f} } \right)_{1} \left( { = \frac{{\tau_{rz} }}{{\left( \rho \right)_{f} \left( {r\Gamma_{1} } \right)^{2} }}} \right), \hfill \\ \left( {C_{f} } \right)_{2} \left( { = \frac{{\tau_{\theta z} }}{{\left( \rho \right)_{f} \left( {r\Gamma_{2} } \right)^{2} }}} \right) \hfill \\ \end{gathered} \right\}, $$

22 $$ \left. \begin{gathered} \left( {Nu_{x} } \right)_{1} \left( { = \frac{{h\left( {q_{w} } \right)}}{{\left( k \right)_{f} \left( {T_{ \circ } - T_{1} } \right)}}} \right), \hfill \\ \left( {Nu_{x} } \right)_{2} \left( { = \frac{{h\left( {q_{w} } \right)}}{{\left( k \right)_{f} \left( {T_{ \circ } - T_{1} } \right)}}} \right) \hfill \\ \end{gathered} \right\}, $$

We obtain the stress $\left( {\tau_{zr} \;{\text{and}}\;\tau_{z\theta } } \right)\,$ and elucidated the shear stresses for the inferior disk with temperature flux $\left( {q_{w} } \right)$.

Skin frictions and Nusselt numbers have non-dimensional versions, which are as follows:

23 $$ \left( {C_{f} } \right)_{1} = \frac{{\left. {\tau_{w} } \right|_{z = 0} }}{{\rho_{f} \left( {r\Gamma_{1} } \right)^{2} }} = \frac{1}{{{\text{Re}}_{r} \left( {1 - \phi } \right)^{2.5} }}\left[ {f^{{\prime\prime}{2}} \left( 0 \right) + g^{{\prime}{2}} \left( 0 \right)} \right]^{\frac{1}{2}} , $$

24 $$ \left( {C_{f} } \right)_{2} = \frac{{\left. {\tau_{w} } \right|_{z = h} }}{{\rho_{f} \left( {r\Gamma_{1} } \right)^{2} }} = \frac{1}{{{\text{Re}}_{r} \left( {1 - \phi } \right)^{2.5} }}\left[ {f^{{\prime\prime}{2}} \left( 1 \right) + g^{{\prime}{2}} \left( 1 \right)} \right]^{\frac{1}{2}} , $$

25 $$ \left. \begin{gathered} Nu_{1} = - \left( {C_{4} + Nr} \right)\theta^{\prime}\left( 0 \right), \hfill \\ Nu_{2} = - \left( {C_{4} + Nr} \right)\theta^{\prime}\left( 1 \right) \hfill \\ \end{gathered} \right\}, $$

Here shear stress $\left( {\tau_{w} } \right)$.

26 $$ \tau_{w} = \sqrt {\tau_{zr}^{2} + \tau_{z\theta }^{2} } $$

## Numerical approach

This section uses MATLAB's bvp4c solver to apply the shooting approach, which is based on the three-stage Lobatto formula with various notable parameters, to numerically solve the velocity and temperature nonlinear ODEs (13–16) with connected reduced boundaries (17). Lobatto-IIIa is a fourth-order accuracy collecting phenomenon. To begin, utilizing innovative considerations for this procedure, higher-order ODEs are converted to ordinary ones. The convergence rate of shooting methods is 10^–6^.

Let,

27 $$ \left. \begin{gathered} f = z_{1} ,f^{\prime\prime} = z_{3} ,f^{\prime} = z_{2} ,f^{\prime\prime\prime} = z^{\prime}_{3} , \hfill \\ g = z_{4} ,\,\,\,\,\,\,\,\,\,\,\,\,\,g^{\prime} = z_{5} ,\,\,\,\,\,\,\,\,g^{\prime\prime} = z^{\prime}_{5} , \hfill \\ p = z_{6} ,\,\,\,\,\,\,\,\,\,\,\,\,\,\,\,\,\,\,\,\,\,\,\,\,\,\,\,\,\,\,\,\,\,\,\,\,p^{\prime} = z^{\prime}_{6} \hfill \\ \theta = z_{7} ,\,\,\,\,\,\,\,\,\,\,\,\,\theta^{\prime} = z_{8} ,\,\,\,\,\,\,\,\,\,\,\theta^{\prime\prime} = z^{\prime}_{8} \hfill \\ \end{gathered} \right\}, $$

28 $$ \left. {z^{\prime}_{3} = \frac{\begin{gathered} {\text{Re}} \beta C_{1} \left( { - 2z_{1} z_{2} z_{3} } \right) + \frac{{C_{1} }}{2}{\text{Re}} \left( { - 2z_{1} z_{3} + z_{2}^{2} - z_{4}^{2} } \right) + \frac{{C_{1} }}{{C_{2} }}Ha\left( {z_{2} + \beta z_{1} z_{3} } \right) + \frac{{C_{1} }}{{C_{2} }} \in \hfill \\ + C_{2} {\text{Re}} Pz_{2} \hfill \\ \end{gathered} }{{\left( {1 - z_{1}^{2} {\text{Re}} \beta C_{1} } \right)}}} \right\}, $$

29 $$ \left. {z^{\prime}_{5} = - C_{1} {\text{Re}} \left( {2z_{1} z_{5} - 2z_{2} z_{4} } \right) + C_{2} {\text{Re}} Pz_{4} + \frac{{C_{1} }}{{C_{2} }}Ha\left( {z_{4} + \beta z_{1} z_{5} } \right)} \right\}, $$

30 $$ \left. {z^{\prime}_{6} = 4C_{2} {\text{Re}} z_{1} z_{2} + 2\frac{{C_{2} }}{{C_{1} }}z_{3} } \right\}, $$

31 $$ \left. {z^{\prime}_{8} = \frac{{4Q_{T} C_{3} {\text{Re}} \left( {z_{1} z_{2} } \right) - 2{\text{Re}} \Pr C_{3} C_{4} z_{1} z_{8} - \Pr Qz_{7} }}{{\left( {\left( {1 + Nr} \right) - 4Q_{T} C_{3} {\text{Re}} z_{1}^{2} } \right)}}} \right\}, $$

With

32 $$ \left. \begin{gathered} \eta = 0,\,\,\,,\,\,\,\,\,\,\,\,\,\,\,\,\,\,\,z_{2} = s_{1} ,\,\,\,\,\,\,\,\,\,\,\,\,\,\,\,\,z_{4} = 1,\,\,\,\,\,\,\,\,\,\,\,\,\,\,\,\,\,\,\,\,\,\,z_{8} = 1, \hfill \\ \frac{{\left( \rho \right)_{thnf} }}{{\left( \rho \right)_{f} }}{\text{Prz}}_{1} + Me\frac{{\left( k \right)_{thnf} }}{{\left( k \right)_{f} }}z_{8} = 0, \hfill \\ \eta = 1,\,\,\,\,\,\,\,\,\,\,\,\,\,\,\,\,z_{2} = s_{2} ,\,\,\,\,\,\,\,\,\,\,\,\,\,\,\,\,\,\,\,\,z_{4} = s_{3} ,\,\,\,\,\,\,\,\,\,\,\,\,\,\,\,\,\,\,\,\,\,z_{8} = 0 \hfill \\ \end{gathered} \right\}. $$

## Result and discussion

**Figure 2:**
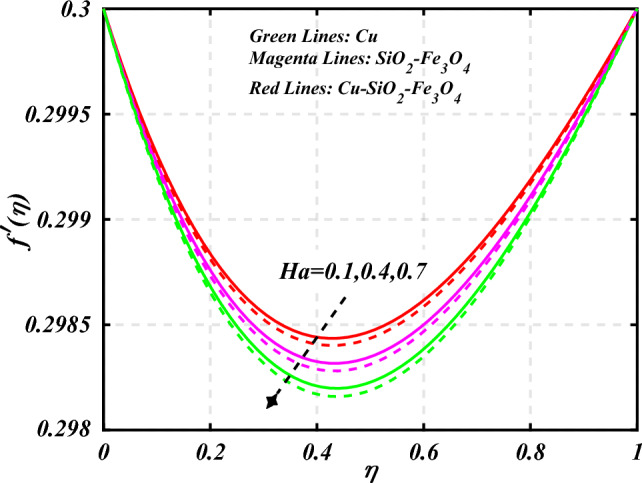
Curve of $Ha$ through $f^{\prime}\left( \eta \right)$.

**Figure 3:**
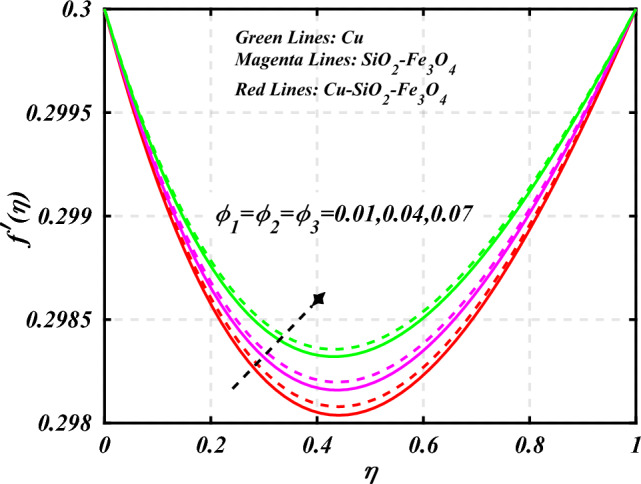
Curve of $\phi_{1} = \phi_{2} = \phi_{3}$ through $f^{\prime}\left( \eta \right)$.

**Figure 4:**
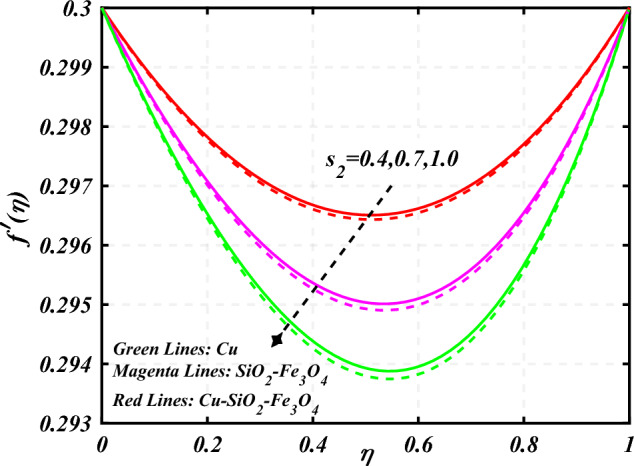
Curve of $s_{2}$ through $f^{\prime}\left( \eta \right)$.

**Figure 5:**
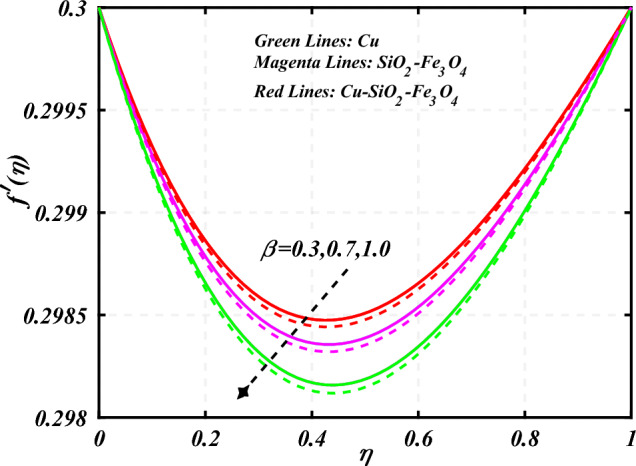
Curve of $\beta$ through $f^{\prime}\left( \eta \right)$.

**Figure 6:**
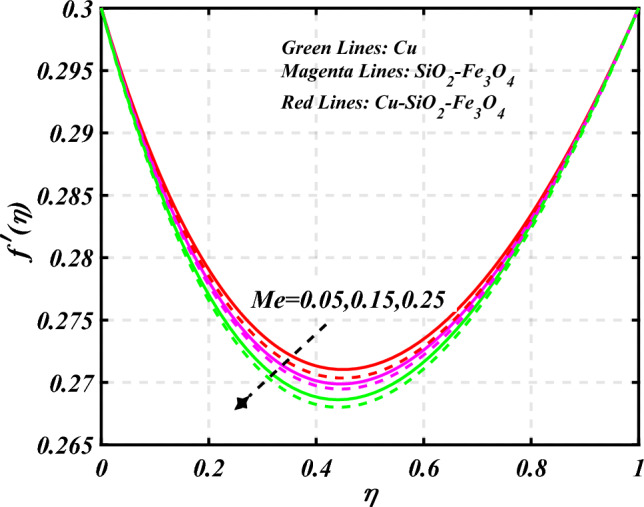
Curve of $Me$ through $f^{\prime}\left( \eta \right)$.

**Figure 7:**
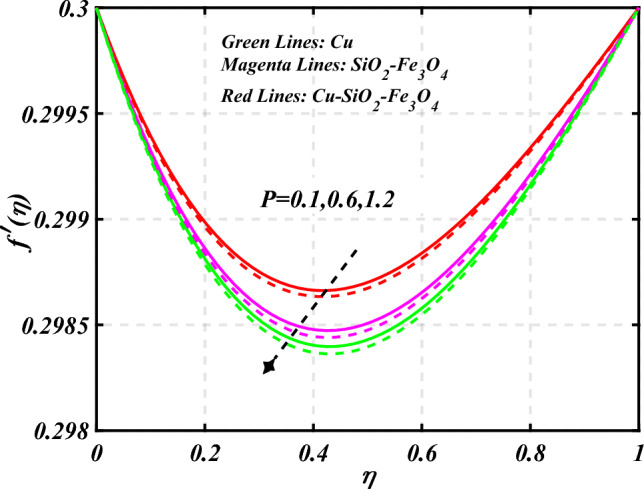
Curve of $P$ through $f^{\prime}\left( \eta \right)$.

**Figure 8:**
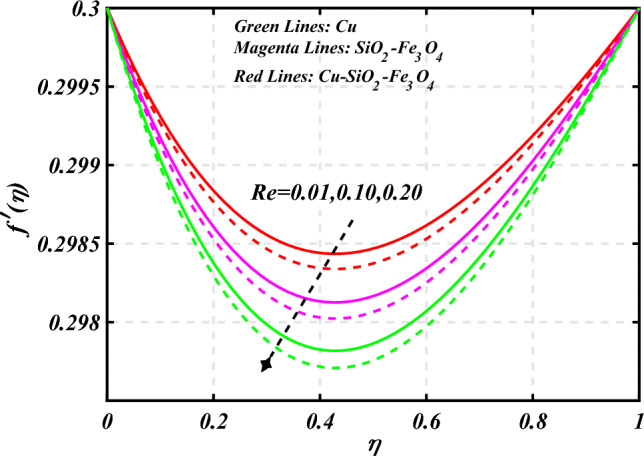
Curve of ${\text{Re}}$ through $f^{\prime}\left( \eta \right)$.

**Figure 9:**
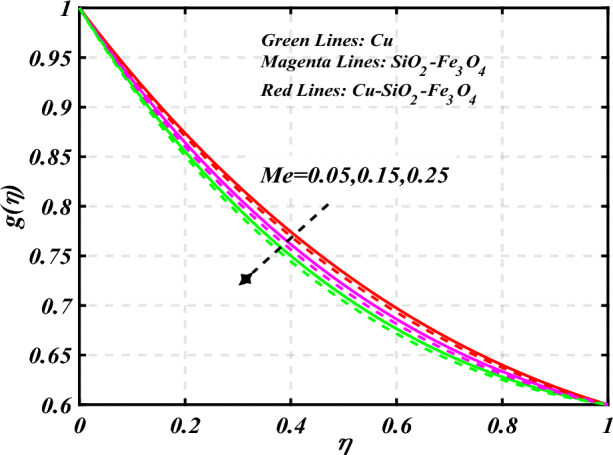
Curve of $Me$ through $g\left( \eta \right)$.

**Figure10:**
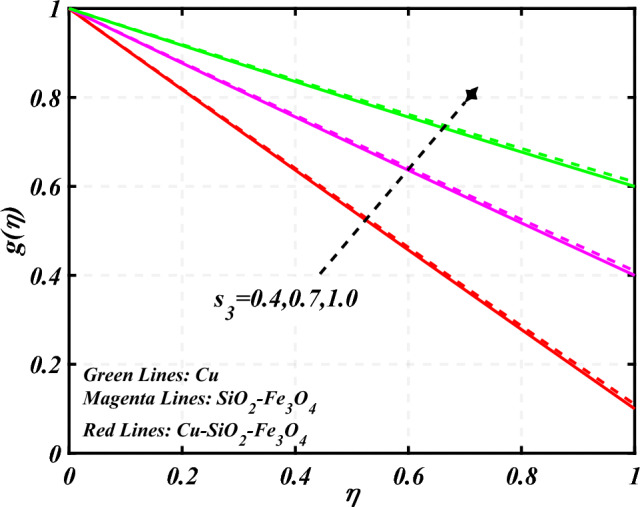
Curve of $s_{3}$ through $g\left( \eta \right)$.

**Figure 11:**
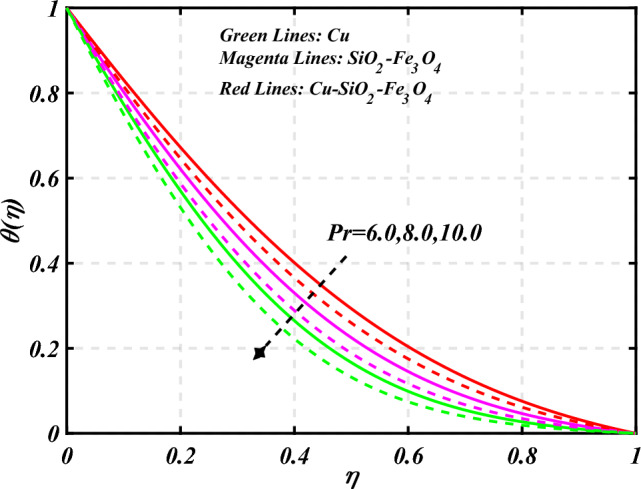
Curve of $Pr$ through $\theta \left( \eta \right)$.

**Figure 12:**
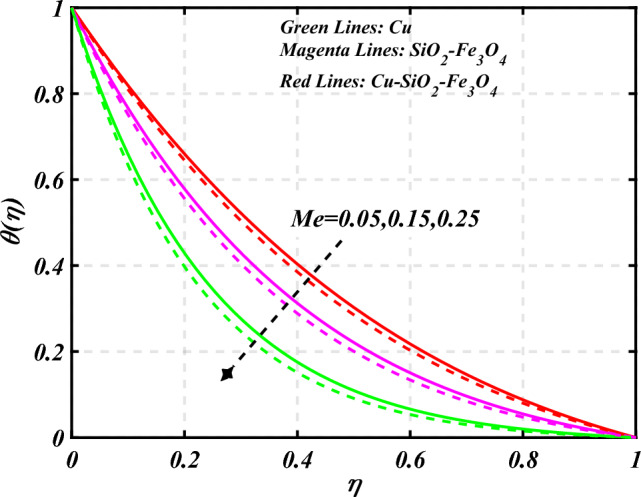
Curve of $Me$ through $\theta \left( \eta \right)$.

**Figure 13:**
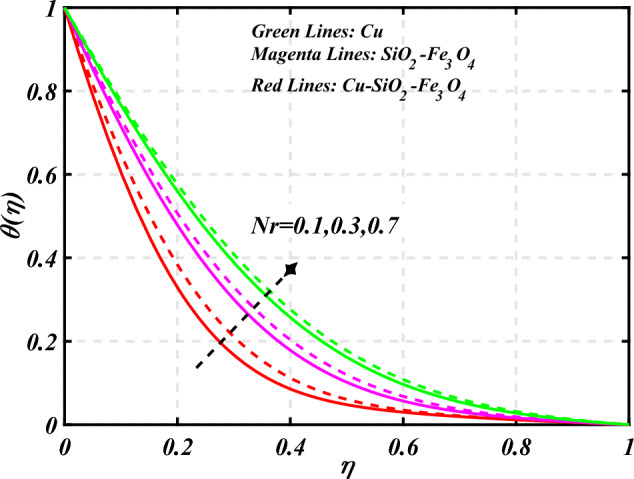
Curve of $Nr$ through $\theta \left( \eta \right)$.

**Figure 14:**
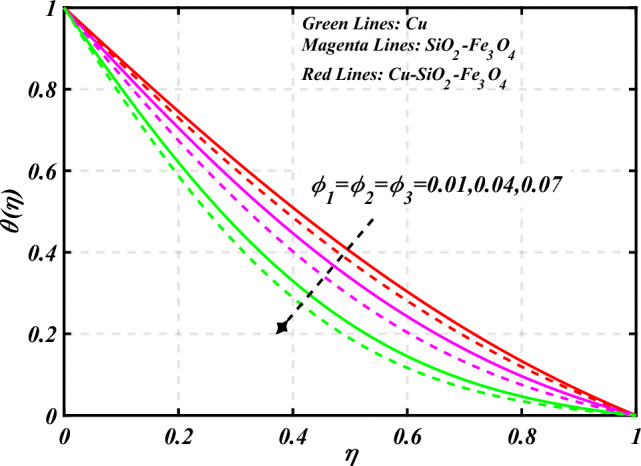
Curve of $\phi_{1} = \phi_{2} = \phi_{3}$ through $\theta \left( \eta \right)$.

**Figure 15:**
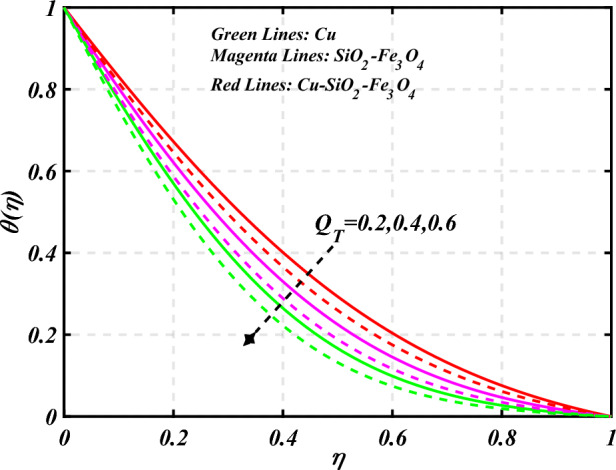
Curve of $Q_{T}$ through $\theta \left( \eta \right)$.

**Figure 16:**
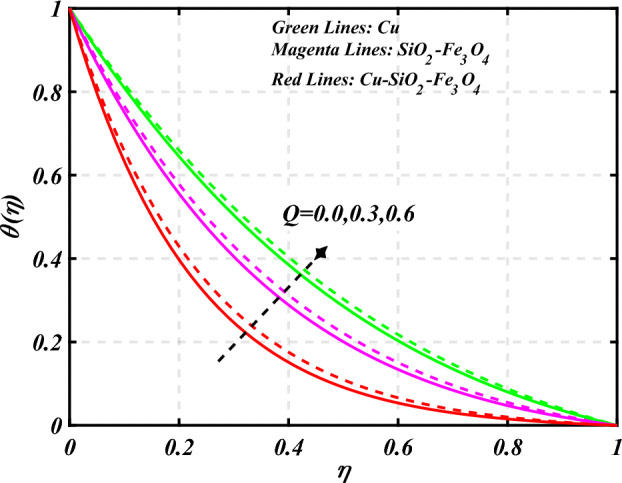
Curve of $Q$ through $\theta \left( \eta \right)$.

**Figure 17:**
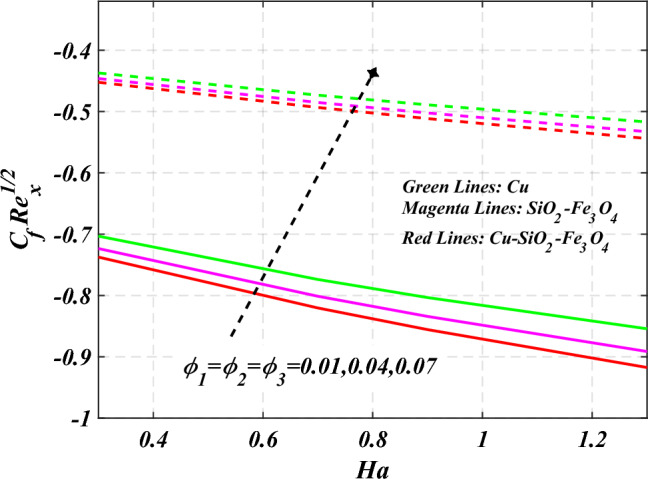
Curve of $\phi_{1} = \phi_{2} = \phi_{3}$ through $C_{f} {\text{Re}}_{x}^{{{1 \mathord{\left/ {\vphantom {1 2}} \right. \kern-0pt} 2}}}$.

**Figure 18:**
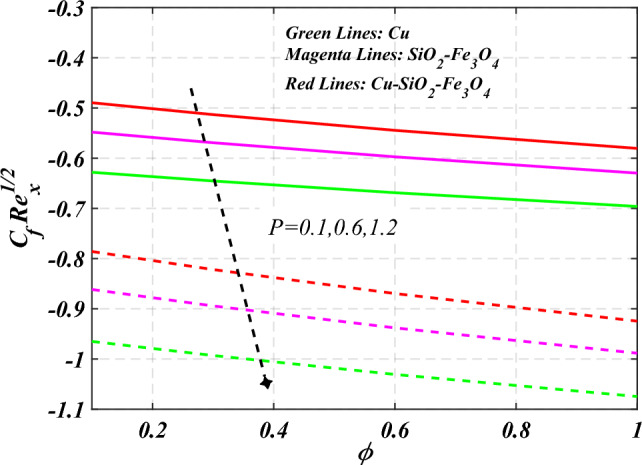
Curve of ${\text{P}} \;\& \;\phi_{1} = \phi_{2} = \phi_{3}$ through $C_{f} {\text{Re}}_{x}^{{{1 \mathord{\left/ {\vphantom {1 2}} \right. \kern-0pt} 2}}}$.

**Figure 19:**
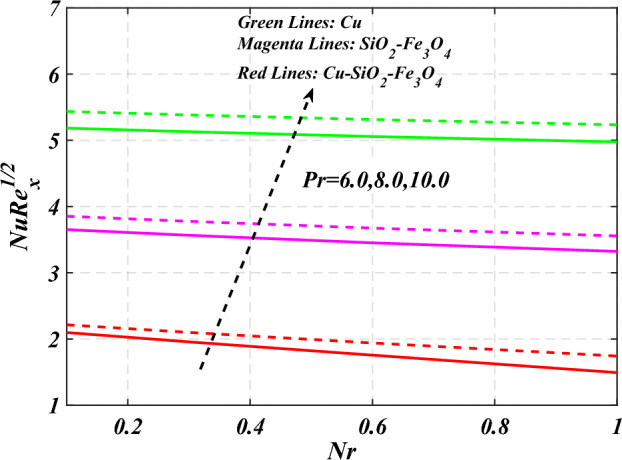
Curve of $Nr\;\& \;\Pr$ through $Nu{\text{Re}}_{x}^{{{1 \mathord{\left/ {\vphantom {1 2}} \right. \kern-0pt} 2}}}$.

**Figure 20:**
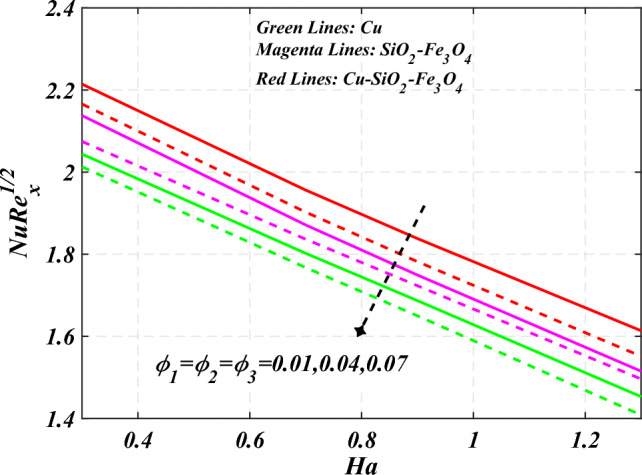
Curve of $Ha\;\& \;\phi_{1} = \phi_{2} = \phi_{3}$ through $Nu{\text{Re}}_{x}^{{{1 \mathord{\left/ {\vphantom {1 2}} \right. \kern-0pt} 2}}}$.

The numerical analysis of Maxwell ternary hybrid nanofluid flow over two discs with MHD, and thermal radiation is performed. A representative set of tangential graphs and temperature distributions for different amounts of relevant parameters. The ranges of the physical parameter are $0.1 < Ha < 0.7, \;\; 0.01 < \phi_{1} = \phi_{2} = \phi_{3} < 0.07, \;\; 0.4 < s_{2} < 1.0,\;\;$ $0.3 < \beta < 1.0,$ $0.05 < Me < 0.25,$ $0.1 < P < 1.2,$ $0.01 < {\text{Re}} < 0.20,$ $0.4 < s_{3} < 1.0,$ $6.0 < \Pr < 10.0,$ $0.1 < Nr < 0.7,$ $0.2 < Q_{T} < 0.6,$ and $0.0 < Q < 0.6$. Figure 2 emphasizes the significance of the Hartmann number $\left( {Ha} \right)$ through the velocity profile $f^{\prime}\left( \eta \right)$. It is noted that the positive values of the Hartmann number $\left( {Ha} \right)$ decline the velocity distribution profile $f^{\prime}\left( \eta \right)$. Physically, a boost in the electromagnetic field enhances the Lorentz force, which reduces the fluid's velocity $f^{\prime}\left( \eta \right)$. The green lines reveal the effects of nanofluid (*Cu/Kerosene oil*), magenta linens shows the aspects of hybrid nanofluid (*SiO*_*2*_*–Fe*_*3*_*O*_*4*_*/Kerosene oil*) and red lines analyzed the importance of ternary hybrid nanofluid (*Cu–SiO*_*2*_*–Fe*_*3*_*O*_*4*_*/Kerosene oil*). Figure 3 investigate the tendency of velocity distribution profile $f^{\prime}\left( \eta \right)$ of nanomaterials for volume fraction of nanoparticles $\left( {\phi_{1} = \phi_{2} = \phi_{3} } \right)$. The $f^{\prime}\left( \eta \right)$ improves for higher volume fraction of nanoparticles $\left( {\phi_{1} = \phi_{2} = \phi_{3} } \right)$. Physically, adding tri-hybrid nano materials to the underlying fluid increases its average viscosity, resulting in such slowdown. The velocity distribution profile $f^{\prime}\left( \eta \right)$ difference through extending parameter at the upper disk $\left( {s_{2} } \right)$ is exhibited in Fig. 4. The velocity rapidly decline when extending parameter at the upper disk higher effect $\left( {s_{2} } \right)$. Figure 5 reveals upshot of fluid parameter $\left( \beta \right)$ on $f^{\prime}\left( \eta \right)$. The higher value of fluid parameter $\left( \beta \right)$ refuses the velocity distribution profile $f^{\prime}\left( \eta \right)$. Figure 6 depicts the consequence of melting parameter $\left( {Me} \right)$ on the velocity distribution profile $f^{\prime}\left( \eta \right)$. Graph indicates that slightly increase in melting parameter $\left( {Me} \right)$ rapidly decline the velocity distribution profile of nanofluid $f^{\prime}\left( \eta \right)$. Therefore, commonly we neglect the applied melting parameter $\left( {Me} \right)$. The green lines reveals the effects of nanofluid (*Cu/Kerosene oil*), magenta linens shows the aspects of hybrid nanofluid (*SiO*_*2*_*–Fe*_*3*_*O*_*4*_*/Kerosene oil*) and red lines analyzed the importance of ternary hybrid nanofluid (*Cu–SiO*_*2*_*–Fe*_*3*_*O*_*4*_*/Kerosene oil*). Figure 7 is displayed for the investigation of velocity distribution profile $f^{\prime}\left( \eta \right)$ against porosity parameter $\left( P \right)$. The velocity distribution profile $f^{\prime}\left( \eta \right)$ of nanofluid increases by increasing the porosity parameter $\left( P \right)$. The effects of Reynolds number $\left( {\text{Re}} \right)$ over velocity distribution profile of nanofluid $f^{\prime}\left( \eta \right)$ in Fig. 8. It witnesses that $f^{\prime}\left( \eta \right)$ of nanofluid turn down for larger variations of Reynolds number $\left( {\text{Re}} \right)$. The influence of melting parameter $\left( {Me} \right)$ over tangential velocity distributions profile $g\left( \eta \right)$ is plotted in the Fig. 9. Due to enhancing the outcomes of melting parameter $\left( {Me} \right)$ the tangential velocity distributions profile $g\left( \eta \right)$ of given nanofluid are downward. The influence of Rotation parameter $\left( {s_{3} } \right)$ over tangential velocity distributions profile $g\left( \eta \right)$ is plotted in the Fig. 10. Due to enhancing the outcomes of Rotation parameter $\left( {s_{3} } \right)$ the tangential velocity distributions profile $g\left( \eta \right)$ of given nanofluid are upward. The green lines reveals the effects of nanofluid (*Cu/Kerosene oil*), magenta linens shows the aspects of hybrid nanofluid (*SiO*_*2*_*–Fe*_*3*_*O*_*4*_*/Kerosene oil*) and red lines analyzed the importance of ternary hybrid nanofluid (*Cu–SiO*_*2*_*–Fe*_*3*_*O*_*4*_*/Kerosene oil*). Figure 11 discussed the aspects of Prandtl number $\left( {\Pr } \right)$ on the thermal profile. The temperature profile $\theta \left( \eta \right)$ boosted up due to the growing outcomes of Prandtl number $\left( {\Pr } \right)$. Physically, increasing Prandtl numbers $\left( {\Pr } \right)$ imply poorer thermal diffusivity, which results in a thinner temperature level of penetration. When the mechanism for transferring heat is supposed to have temperature dependent thermal conductivity, the field of heat has a greater value. Figure 12 described the influence of melting parameter $\left( {Me} \right)$ over thermal distributions profile $\theta \left( \eta \right)$. The temperature of given nanofluid is decline due to the increasing values of melting parameter $\left( {Me} \right)$. The green lines reveals the effects of nanofluid (*Cu/Kerosene oil*), magenta linens shows the aspects of hybrid nanofluid (*SiO*_*2*_*–Fe*_*3*_*O*_*4*_*/Kerosene oil*) and red lines analyzed the importance of ternary hybrid nanofluid (*Cu–SiO*_*2*_*–Fe*_*3*_*O*_*4*_*/Kerosene oil*).The effect of thermal radiation parameter over temperature $\theta \left( \eta \right)$ is illustrated in the Fig. 13. This graph demonstrates that the increasing value of thermal radiation parameter $\left( {Nr} \right)$ improves the temperature profile $\theta \left( \eta \right)$. The addition of thermal radiation parameter $\left( {Nr} \right)$ to the temperature $\theta \left( \eta \right)$ improves fluids randomized mobility. As a result of the constant impact, additional heat is produced. Figure 14 analyzed the effects of temperature profile $\theta \left( \eta \right)$ for the various outcomes of volume fraction of nanoparticles $\left( {\phi_{1} = \phi_{2} = \phi_{3} } \right)$. The temperature is declined for the increasing variations of $\left( {\phi_{1} = \phi_{2} = \phi_{3} } \right)$. Figure 15 explore that the importance of thermal relaxation parameter $\left( {Q_{T} } \right)$ on the thermal distributions profile. The increasing variations of thermal relaxation parameter $\left( {Q_{T} } \right)$ decline the thermal distributions profile $\theta \left( \eta \right)$. The green lines reveals the effects of nanofluid (*Cu/Kerosene oil*), magenta linens shows the aspects of hybrid nanofluid (*SiO*_*2*_*–Fe*_*3*_*O*_*4*_*/Kerosene oil*) and red lines analyzed the importance of ternary hybrid nanofluid (*Cu-SiO*_*2*_*–Fe*_*3*_*O*_*4*_*/Kerosene oil*). The effect of heat source sink parameter $\left( Q \right)$ over temperature $\theta \left( \eta \right)$ is illustrated in the Fig. 16. This graph demonstrates that the increasing value of $\left( Q \right)$ improves the $\theta \left( \eta \right)$. The green lines reveals the effects of nanofluid (*Cu/Kerosene oil*), magenta linens shows the aspects of hybrid nanofluid (*SiO*_*2*_*–Fe*_*3*_*O*_*4*_*/Kerosene oil*) and red lines analyzed the importance of ternary hybrid nanofluid (*Cu–SiO*_*2*_*–Fe*_*3*_*O*_*4*_*/Kerosene oil*). Figure 17 symbolize the effects volume fraction of nanoparticles $\left( {\phi_{1} = \phi_{2} = \phi_{3} } \right)$ and magnetic parameter $\left( {Ha} \right)$ over $\left( {C_{f} {\text{Re}}_{x}^{{{1 \mathord{\left/ {\vphantom {1 2}} \right. \kern-0pt} 2}}} } \right)$. The $\left( {C_{f} {\text{Re}}_{x}^{{{1 \mathord{\left/ {\vphantom {1 2}} \right. \kern-0pt} 2}}} } \right)$ is augments as $\left( {\phi_{1} = \phi_{2} = \phi_{3} } \right)$ the increase. It is to be renowned that $\left( {C_{f} {\text{Re}}_{x}^{{{1 \mathord{\left/ {\vphantom {1 2}} \right. \kern-0pt} 2}}} } \right)$ is larger estimations. Figure 18 denotes the consequence of $\left( {C_{f} {\text{Re}}_{x}^{{{1 \mathord{\left/ {\vphantom {1 2}} \right. \kern-0pt} 2}}} } \right)$ for $\left( {\phi_{1} = \phi_{2} = \phi_{3} } \right)$ and $\left( P \right)$. It is examined that $\left( {C_{f} {\text{Re}}_{x}^{{{1 \mathord{\left/ {\vphantom {1 2}} \right. \kern-0pt} 2}}} } \right)$ is raise with the augmentation in the variations of porosity parameter. Figure 19 reveal the impact of $\left( {Nu{\text{Re}}_{x}^{{{1 \mathord{\left/ {\vphantom {1 2}} \right. \kern-0pt} 2}}} } \right)$ when the $\left( {Nr} \right)$ and $\left( {\Pr } \right)$ are supplied with dissimilar inputs. The $\left( {Nu{\text{Re}}_{x}^{{{1 \mathord{\left/ {\vphantom {1 2}} \right. \kern-0pt} 2}}} } \right)$ endures an important decrease with the thermal radiation $\left( {Nr} \right)$ and Prandtl number $\left( {\Pr } \right)$ enhance. Figure 20 represents the fluctuation $\left( {Nu{\text{Re}}_{x}^{{{1 \mathord{\left/ {\vphantom {1 2}} \right. \kern-0pt} 2}}} } \right)$ with the motivation of the volume fraction of nanoparticles and Hartmann number. It has been showing that the local Nusselt number $\left( {Nu{\text{Re}}_{x}^{{{1 \mathord{\left/ {\vphantom {1 2}} \right. \kern-0pt} 2}}} } \right)$ enlarges as volume fraction of nanoparticles $\left( {\phi_{1} = \phi_{2} = \phi_{3} } \right)$ raise. Table 1 analyzed the thermophysical features of nanofluid, hybrid nanofluid, and ternary hybrid nanofluid. Table 2 reveals the physical properties of solid particles and base liquid. Table 3 analyzed the **c**omparison of numerical outcomes for $s_{3}$ through $f^{\prime\prime}\left( 0 \right)$. It demonstrates the good agreement between the old and present frameworks.

**Table 1:** Nanofluid, hybrid nanofluid, and ternary hybrid nanofluid thermophysical characteristics^3,39,40^.

Properties	Nanofluid
Density $\left( {\rho_{nf} } \right)$	$\rho_{nf} \left( { = \rho_{f} \left( {1 - \phi_{1} } \right) + \phi_{1} \left( {\rho_{{s_{1} }} } \right)} \right)$
Viscosity $\left( {\mu_{nf} } \right)$	$\mu_{nf} \left( { = \frac{{\mu_{f} }}{{\left( {1 - \phi_{1} } \right)^{2.5} }}} \right)$
Heat capacity $\left( {\rho C_{p} } \right)_{nf}$	$$\left( {\rho C_{p} } \right)_{nf} \left( \begin{gathered} = \left( {\rho C_{p} } \right)_{f} \left( {1 - \phi_{1} } \right) \hfill \\ + \phi_{1} \left( {\rho C_{p} } \right)_{{s_{1} }} \hfill \\ \end{gathered} \right)$$
Thermal conductivity $\left( {K_{nf} } \right)$	$\frac{{K_{nf} }}{{K_{f} }}\left( { = \frac{{K_{{s_{1} }} + 2K_{f} - 2\left( {K_{f} - K_{{s_{1} }} } \right)\phi_{1} }}{{K_{{s_{1} }} + 2K_{f} + \phi_{1} \left( {K_{f} - K_{{s_{1} }} } \right)}}} \right)$

Properties	Hybrid nanofluid
Density $\left( {\rho_{hnf} } \right)$	$$\rho_{hnf} \left( \begin{gathered} = \left( {1 - \phi_{2} } \right)\left( \begin{gathered} \left( {1 - \phi_{1} } \right)\rho_{f} \hfill \\ + \phi_{1} \rho_{s1} \hfill \\ \end{gathered} \right) \hfill \\ + \phi_{2} \rho_{s2} \hfill \\ \end{gathered} \right)$$
Viscosity $\left( {\mu_{hnf} } \right)$	$\mu_{hnf} \left( { = \frac{{\mu_{f} }}{{\left( {1 - \phi_{1} } \right)^{2.5} \left( {1 - \phi_{2} } \right)^{2.5} }}} \right)$
Heat capacity $\left( {\rho C_{p} } \right)_{hnf}$	$$\left( {\rho C_{p} } \right)_{hnf} \left( { = \left( {1 - \phi_{2} } \right)\left( \begin{gathered} \left( {1 - \phi_{1} } \right)\left( {\rho C_{p} } \right)_{f} \hfill \\ + \phi_{1} \left( {\rho C_{p} } \right)_{s1} \hfill \\ \end{gathered} \right) + \phi_{2} \left( {\rho C_{p} } \right)_{s2} } \right)$$
Thermal conductivity $\left( {K_{hnf} } \right)$	$$\begin{gathered} \frac{{K_{hnf} }}{{K_{nf} }}\left( { = \frac{{K_{s2} + 2K_{nf} - 2\left( {K_{nf} - K_{s2} } \right)\phi_{2} }}{{K_{s2} + 2K_{nf} + \phi_{2} \left( {K_{nf} - K_{s2} } \right)}}} \right), \hfill \\ Here \hfill \\ \,\frac{{K_{nf} }}{{K_{f} }}\left( { = \frac{{K_{s1} + 2K_{f} - 2\left( {K_{f} - K_{s1} } \right)\phi_{1} }}{{K_{s1} + 2K_{f} + \phi_{1} \left( {K_{f} - K_{s1} } \right)}}} \right) \hfill \\ \end{gathered}$$

Properties	Hybrid nanofluid
Density $\left( {\rho_{thnf} } \right)$	$$\rho_{thnf} \left( { = \left( {1 - \phi_{3} } \right)\left( \begin{gathered} \left( {1 - \phi_{2} } \right)\left[ {\left( {1 - \phi_{1} } \right)\rho_{f} + \phi_{1} \rho_{s1} } \right] \hfill \\ + \phi_{2} \rho_{s2} \hfill \\ \end{gathered} \right) + \phi_{3} \rho_{s3} } \right)$$
Viscosity $\left( {\mu_{thnf} } \right)$	$\mu_{thnf} \left( { = \frac{{\mu_{f} }}{{\left( {1 - \phi_{1} } \right)^{2.5} \left( {1 - \phi_{2} } \right)^{2.5} \left( {1 - \phi_{3} } \right)^{2.5} }}} \right)$
Heat capacity $\left( {\rho C_{p} } \right)_{thnf}$	$$\left( {\rho C_{p} } \right)_{thnf} \left( { = \left( {1 - \phi_{3} } \right)\left( \begin{gathered} \left( {1 - \phi_{2} } \right)\left( \begin{gathered} \left( {1 - \phi_{1} } \right)\left( {\rho C_{p} } \right)_{f} \hfill \\ + \phi_{1} \left( {\rho C_{p} } \right)_{s1} \hfill \\ \end{gathered} \right) \hfill \\ + \phi_{2} \left( {\rho C_{p} } \right)_{s2} \hfill \\ \end{gathered} \right) + \phi_{3} \left( {\rho C_{p} } \right)_{s3} } \right)$$
Thermal conductivity $\left( {K_{thnf} } \right)$	$$\begin{gathered} \frac{{K_{thnf} }}{{K_{hnf} }}\left( { = \frac{{K_{s3} + 2K_{hnf} - 2\left( {K_{hnf} - K_{s3} } \right)\phi_{3} }}{{K_{s3} + 2K_{hnf} + \phi_{3} \left( {K_{hnf} - K_{s3} } \right)}}} \right), \hfill \\ \frac{{K_{hnf} }}{{K_{nf} }}\left( { = \frac{{K_{s2} + 2K_{nf} - 2\left( {K_{nf} - K_{s2} } \right)\phi_{2} }}{{K_{s2} + 2K_{nf} + \phi_{2} \left( {K_{nf} - K_{s2} } \right)}}} \right), \hfill \\ and \hfill \\ \,\frac{{K_{nf} }}{{K_{f} }}\left( { = \frac{{K_{s1} + 2K_{f} - 2\left( {K_{f} - K_{s1} } \right)\phi_{1} }}{{K_{s1} + 2K_{f} + \phi_{1} \left( {K_{f} - K_{s1} } \right)}}} \right) \hfill \\ \end{gathered}$$

**Table 2:** Physical features of solid particles and base liquid^41–44^.

Nanoparticles and base fluid	$C_{p} \;\;\left( {\frac{{\text{J}}}{kg\;K}} \right)$	$\rho \;\;\left( {\frac{{{\text{kg}}}}{{{\text{m}}^{{3}} }}} \right)$	$k\;\;\left( {\frac{{\text{W}}}{{\text{m}}}} \right)$	$\sigma \;\;\left( {\frac{{\text{S}}}{{\text{m}}}} \right)$
*Fe*_*3*_*O*_*4*_	670	5200	6	25,000
*Cu*	385	8933	402	$5.96 \times 10^{7}$
*SiO*_*2*_	703	2200	1.2	–
*Kerosene oil*	2090	783	0.15	–

**Table 3:** The $s_{3}$ through $f^{\prime\prime}\left( 0 \right)$ numerical outputs are compared.

Parameter	Turkyilmazoglu^45^	Kumar et al.^46^	Present outcomes
$s_{3}$	$f^{\prime\prime}\left( 0 \right)$	$f^{\prime\prime}\left( 0 \right)$	$f^{\prime\prime}\left( 0 \right)$
-1.0	0.06665317	0.06665332	0.06665334
-0.7	0.08394209	0.08393221	0.08393238
-0.4	0.10394091	0.10394119	0.10394151
0.0	0.09996226	0.09996273	0.09996290
0.4	0.06663425	0.06663453	0.06663472

## Conclusion

The Cattaneo-Christov heat flux diffusion theory is used to analyze the ternary hybrid nano liquid over double discs. The main PDEs are converted into ODEs by using similarities transformations. The ODEs are numerically solved using the bvp4c solver in the computational tool MATLAB. The present work's main points are as follows:

- The velocity profile id reduced as the fluid parameter and Reynolds number increased.
- The velocity profile boosted up for the rotation parameter while declined for the porosity parameter.
- The both velocities of nanofluid are decline by improving outcomes of melting parameter.
- Increase the temperature of the nanofluid to improve the findings of the thermal radiation parameter and the heat source sink parameter.
- As the Prandtl number and thermal relation parameter increase, the temperature of the flowing fluid decreases.

## Data Availability

The datasets used and analyzed during the current study are available from the corresponding author upon reasonable request.

## List of symbols

$\left( {u,v,w} \right)$: Components of velocity $\left( {{\text{m}}\;{\text{s}}^{ - 1} } \right)$
$$\left( {r,z} \right)$$: Coordinates
$\left( {\Gamma_{1} } \right)$: The upper disk rotates with angular velocity $\left( {{\text{m}}\;{\text{s}}^{ - 1} } \right)$
$\left( {\Gamma_{2} } \right)$: The lower disk rotates with angular velocity $\left( {{\text{m}}\;{\text{s}}^{ - 1} } \right)$
$\left( h \right)$: Distance between the disks
$\left( {\phi_{1} = \phi_{2} = \phi_{3} } \right)$: Nanoparticles volume fraction
$$\left( {Ha} \right)$$: Magnetic parameter
$$\left( {\text{Re}} \right)$$: Reynolds number
$$\left( {Nr} \right)$$: Thermal radiation
$$\left( {Pr} \right)$$: Prandtl number
$$\left( {Me} \right)$$: Melting parameter
$\left( {s_{1} } \right)$: Extending stricture at the lower disk
$\left( {s_{2} } \right)$: Extending parameter at the upper disk
$$\left( {s_{3} } \right)$$: Rotation parameter
$\left( Q \right)$: Heat source-sink parameter
$\left( {Q_{T} } \right)$: Thermal relaxation parameter
$$\left( {C_{1} } \right)$$: Constant
$$\left( {C_{2} } \right)$$: Constant
$$\left( {C_{3} } \right)$$: Constant
$$\left( {C_{4} } \right)$$: Constant
$\left( {\tau_{w} } \right)$: Skin Friction or Shear Stress
$\left( {K_{nf} } \right)$: Thermal conductivity of nanofluid $\left( {{\text{W}}\;{\text{m}}^{ - 1} \;{\text{K}}^{ - 1} } \right)$
$\left( {\rho C_{p} } \right)_{nf}$: Heat capacity of nanofluid $\left( {{\text{J}}\;{\text{m}}^{ - 3} \;{\text{K}}^{ - 1} } \right)$
$\left( {\mu_{nf} } \right)$: Viscosity of nanofluid $\left( {{\text{kg}}\;{\text{m}}^{ - 1} {\text{s}}^{ - 1} } \right)$
$\left( {\rho_{nf} } \right)$: Density of nanofluid $\left( {{\text{kg}}\;{\text{m}}^{ - 3} } \right)$
$\left( {K_{hnf} } \right)$: Thermal conductivity of hybrid nanofluid $\left( {{\text{W}}\;{\text{m}}^{ - 1} \;{\text{K}}^{ - 1} } \right)$
$\left( {\rho C_{p} } \right)_{hnf}$: Heat capacity of hybrid nanofluid $\left( {{\text{J}}\;{\text{m}}^{ - 3} {\text{K}}^{ - 1} } \right)$
$\left( {\mu_{hnf} } \right)$: The viscosity of hybrid nanofluid $\left( {{\text{kg}}\;{\text{m}}^{ - 1} \;{\text{s}}^{ - 1} } \right)$
$\left( {\rho_{hnf} } \right)$: The density of hybrid nanofluid $\left( {{\text{kg}}\;{\text{m}}^{ - 3} } \right)$
$\left( {\mu_{thnf} } \right)$: The viscosity of ternary hybrid nanofluid $\left( {{\text{kg}}\;{\text{m}}^{ - 1} \;{\text{s}}^{ - 1} } \right)$
$\left( {K_{thnf} } \right)$: Thermal conductivity of ternary hybrid nanofluid $\left( {{\text{W}}\;{\text{m}}^{ - 1} \;{\text{K}}^{ - 1} } \right)$
$\left( {\rho C_{p} } \right)_{thnf}$: Heat capacity of ternary hybrid nanofluid $\left( {{\text{J}}\;{\text{m}}^{ - 3} \;{\text{K}}^{ - 1} } \right)$
$\left( {\rho_{thnf} } \right)$: The density of ternary hybrid nanofluid $\left( {{\text{kg}}\;{\text{m}}^{ - 3} } \right)$
